# Secretagogin is a Ca^2+^-dependent stress-responsive chaperone that may also play a role in aggregation-based proteinopathies

**DOI:** 10.1016/j.jbc.2022.102285

**Published:** 2022-07-21

**Authors:** Amrutha H. Chidananda, Radhika Khandelwal, Aditya Jamkhindikar, Asmita D. Pawar, Anand K. Sharma, Yogendra Sharma

**Affiliations:** 1 CSIR-Centre for Cellular and Molecular Biology (CCMB), Hyderabad, India; 2 Academy of Scientific and Innovative Research (AcSIR), Ghaziabad, India; 3 Department of Biological Sciences, Indian Institute of Science Education and Research (IISER), Berhampur, India

**Keywords:** secretagogin, protein misfolding, neurodegenerative disease, stress-responsive protein, Ca^2+^-dependent chaperone, ADH, alcohol dehydrogenase, CS, citrate synthase, ΔCTD, C-terminal deleted, HSP70, heat shock protein 70, ITC, isothermal titration calorimetry, MDH, malate dehydrogenase, ΔNTD, N-terminal deleted, SCGN, secretagogin, SAXS, small-angle X-ray scattering

## Abstract

Secretagogin (SCGN) is a three-domain hexa-EF-hand Ca^2+^-binding protein that plays a regulatory role in the release of several hormones. SCGN is expressed largely in pancreatic β-cells, certain parts of the brain, and also in neuroendocrine tissues. The expression of SCGN is altered in several diseases, such as diabetes, cancers, and neurodegenerative disorders; however, the precise associations that closely link SCGN expression to such pathophysiologies are not known. In this work, we report that SCGN is an early responder to cellular stress, and SCGN expression is temporally upregulated by oxidative stress and heat shock. We show the overexpression of SCGN efficiently prevents cells from heat shock and oxidative damage. We further demonstrate that in the presence of Ca^2+^, SCGN efficiently prevents the aggregation of a broad range of model proteins *in vitro*. Small-angle X-ray scattering (BioSAXS) studies further reveal that Ca^2+^ induces the conversion of a closed compact apo-SCGN conformation into an open extended holo-SCGN conformation *via* multistate intermediates, consistent with the augmentation of chaperone activity of SCGN. Furthermore, isothermal titration calorimetry establishes that Ca^2+^ enables SCGN to bind α-synuclein and insulin, two target proteins of SCGN. Altogether, our data not only demonstrate that SCGN is a Ca^2+^-dependent generic molecular chaperone involved in protein homeostasis with broad substrate specificity but also elucidate the origin of its altered expression in several cancers. We describe a plausible mechanism of how perturbations in Ca^2+^ homeostasis and/or deregulated SCGN expression would hasten the process of protein misfolding, which is a feature of many aggregation-based proteinopathies.

Secretagogin (SCGN) is a 32 kDa Ca^2+^-binding protein of the hexa EF-hand family, which was first discovered in the cDNA library of human pancreatic β-cells as a facilitator of insulin secretion (1). It is highly enriched in pancreatic islets and well-characterized for its role in glucose metabolism by regulating insulin expression and release through SNAP25 interaction (1, 2, 3). SCGN is also expressed in neuroendocrine cells of gastrointestinal tract and certain brain tissues. Deregulated SCGN expression is reported in a broad range of disorders that include diabetes (4, 5, 6), neurodegenerative disorders (7, 8, 9), cancer (10, 11, 12), and ulcerative colitis (13). The consistent deregulated expression of SCGN leads to the proposition that SCGN could be considered a potential biomarker for certain cancers, such as prostate, large cell neuroendocrine, pituitary, colorectal, and renal cancers (10, 11, 14, 15, 16, 17, 18).

Besides insulin, SCGN also regulates the secretion of stress-related corticotrophin-releasing hormone (19) and matrix metalloprotease-2 from selective neurons (20). Thus, SCGN is a multifunctional regulator of several hormones and is also emerging as a stress-related Ca^2+^ sensor (19, 21, 22). Despite such strong correlations, the functions and mechanistic details are not understood. Recent reports have also illustrated the role of SCGN in preventing insulin and α-synuclein aggregation (6, 23). Protein misfolding leads to the onset of cellular stress, which, in various pathological conditions, leads to protein aggregation. These findings prompted us to explore the possibility that SCGN could be involved in managing cellular stress by preventing protein misfolding.

In this study, we demonstrate that SCGN is a Ca^2+^-dependent molecular chaperone. We report a connection between SCGN expression and cellular stress; SCGN is temporally upregulated during heat and oxidative stress, suggestively alleviating the aggregation of target proteins and mitigating cell death. This finding underscores the strong association of SCGN with ER stress and the redox-responsive Ca^2+^ switch of SCGN (5, 24), which would aid in regulating SCGN function both in the ER (oxidizing) and the cytosol (reducing). Using the BioSAXS, we further demonstrate that the Ca^2+^-dependent chaperone activity of SCGN is associated with its transformation from closed to open conformation upon Ca^2+^ binding. Thus, we identify a new role of SCGN as a Ca^2+^-dependent general chaperone and suggest its larger, yet unappreciated, involvement in precluding protein misfolding disorders.

## Results

### SCGN expression is altered by heat and oxidative stress

To discern a correlation of SCGN expression with protein misfolding diseases, we first looked for alterations in the temporal expression of endogenous SCGN under stress conditions. Change in cellular SCGN expression during stress was monitored by subjecting RIN-5F cells to heat and oxidative stresses. As a standard procedure, heat shock was induced by incubating cells at 42 °C (25, 26), while oxidative stress was induced chemically by treating the cells with rotenone, a mitochondria complex I inhibitor known to induce constitutive protein aggregation *in vitro*. The endogenous SCGN expression was monitored at both the transcript and protein levels, at different time points after exposure to stress.

**Figure 1:**
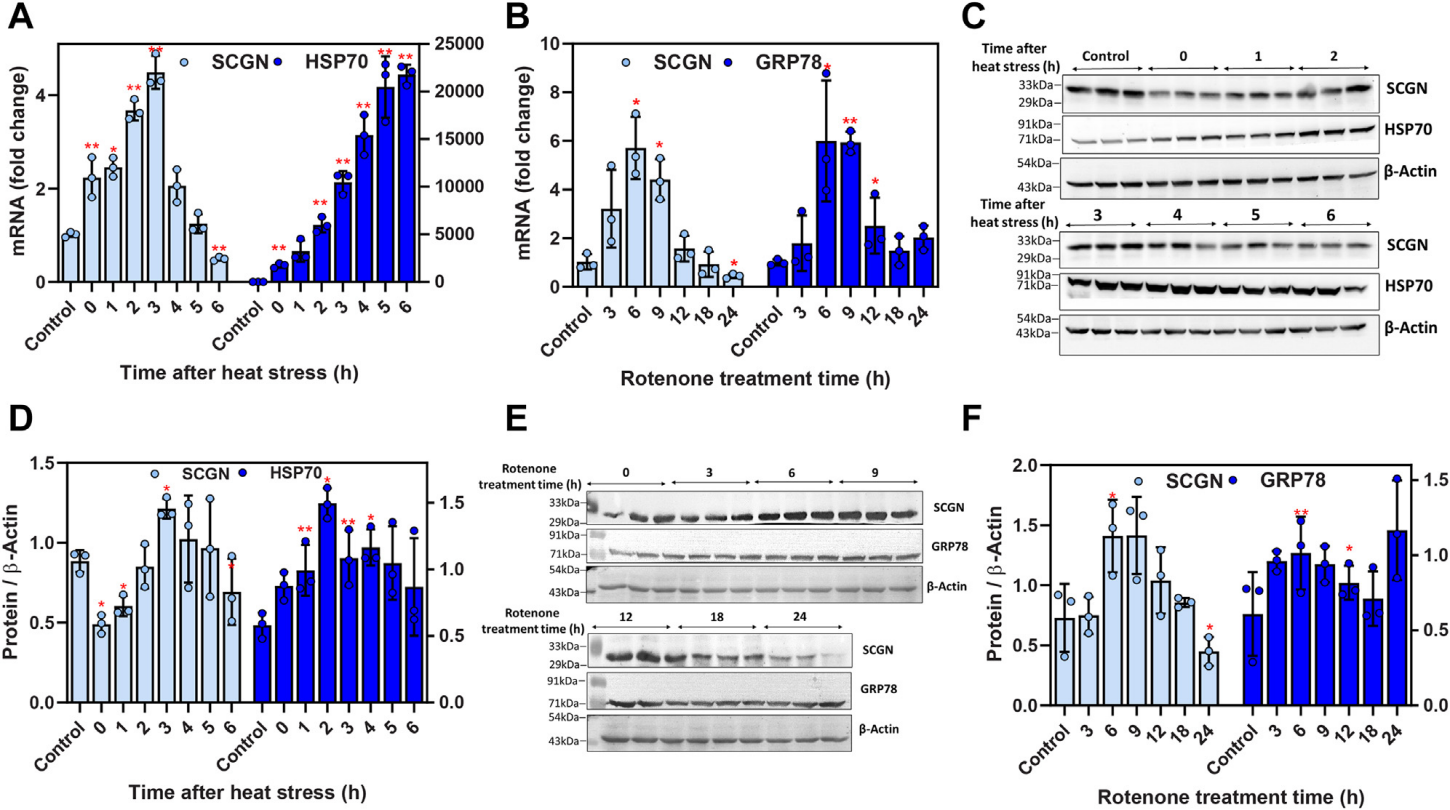
**SCGN expression is altered during heat and oxidative stress.***A*, transcript levels of SCGN in RIN-5F cells that were subjected to heat shock treatment at 42 °C for 45 min and then lysed after 0, 1, 2, 3, 4, 5, and 6 h of the recovery period. SCGN mRNA levels as analyzed by qRT-PCR. *B*, SCGN mRNA levels in RIN-5F cells after rotenone treatment (1 μM) for 3, 6, 9, 12, 18, and 24 h. *C*, Western blot depicting SCGN levels in RIN-5F cells subjected to heat shock treatment as in (*A*), *E*, Western blot depicting SCGN protein levels in RIN-5F cells treated with rotenone at 3, 6, 9, 12, 18, and 24 h of time points, and *(D* and *F*) quantitative analyses of immunoblots showing signal intensity performed using ImageJ. Signal intensity is normalized with β-actin. HSP70 and GRP78 were used as positive controls for qRT-PCR and western blots in heat and oxidative stress, respectively. ∗ Represents *p*-value <0.05 and ∗∗represents *p*-value <0.01 with a minimum of three biological replicates in a group. HSP70, heat shock protein 70; SCGN, secretagogin.

A maximum of a 4-fold increase in *Scgn* gene expression was observed 3 h postshock treatment (Fig. 1*A*). After steadily increasing for 3 h postheat shock treatment, a gradual decline in *Scgn* expression was observed till 6 h. The expression of inducible heat shock protein 70 (*Hsp70*), which was used as a positive control was found to be maximum at 6 h which coincides with the previous reports (27, 28). This suggests that SCGN is an early responder to heat shock. A similar pattern was noted in rotenone-treated cells, where *Scgn* transcript remains significantly upregulated between 6 to 9 h followed by a retreat to below normal levels (Fig. 1*B*). This was compared with glucose-regulated protein (*Grp78*) levels, a known marker for oxidative stress (29), whose expression was found elevated around 6 to 9 h as demonstrated by quantitative real-time PCR (qRT-PCR) analyses. These results demonstrate significant upregulation of *Scgn* transcripts by thermal or oxidative stress.

To check if the change in transcript level also translates to a change in SCGN protein levels, we examined SCGN levels after heat shock and rotenone treatment. A noticeable increase in SCGN levels, similar to that seen in HSP70 expression, was seen until 6 h of recovery after heat shock treatment (Fig. 1, *C* and *D*). Oxidative stress induced by rotenone treatment resulted in an increase in SCGN protein levels for up to 9 h, followed by a significant reduction in the protein levels (Fig. 1, *E* and *F*). Consistent with the transcript level, GRP78 protein level was found to be upregulated 6 h post rotenone treatment. The transient increase in transcript levels of SCGN during the initial short-term recovery phase after heat shock or oxidative stress is followed by rapid transcript degradation (Fig. 1, *A* and *B*). Likewise, the SCGN protein levels elevate during the early response to both heat shock and oxidative stress, but during the late response to oxidative stress, SCGN is maintained at a lower steady-state level (Fig. 1, *D* and *F*). This indicates that SCGN is an early responder to stress.

### SCGN suppresses the aggregation of proteins in a Ca^2+^-dependent manner

**Figure 2:**
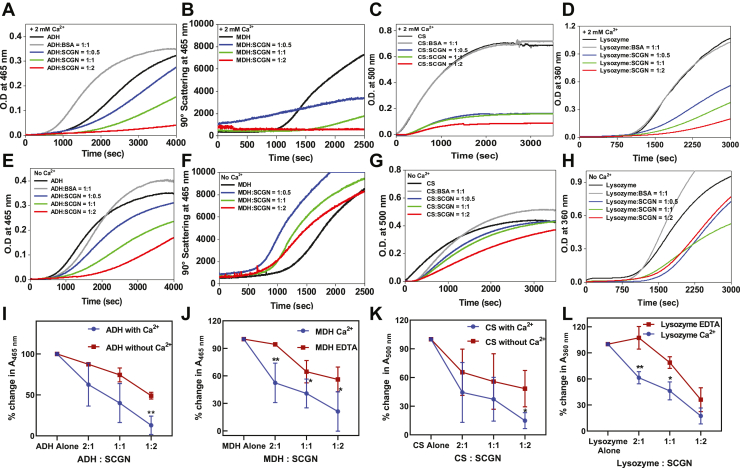
**SCGN prevents aggregation of various model substrates**. Aggregation assay of substrates: alcohol dehydrogenase (ADH), malate dehydrogenase (MDH), citrate synthase (CS), and lysozyme in the presence of SCGN at different stoichiometric ratios. Bovine serum albumin (BSA) was used as a control. The aggregation kinetics was monitored in the presence of either 2 mM Ca^2+^: (*A*) ADH, (*B*) MDH, (*C*) CS, (*D*) lysozyme, or in the presence of 10 μM EDTA: (*E*) ADH, (*F*) MDH, (*G*) CS, and (*H*) lysozyme. Percentage change in chaperone activity of SCGN in the presence of 10 μM EDTA or 2 mM Ca^2+^ with: (*I*) ADH, (*J*) MDH, (*K*) CS, and (*L*) lysozyme, at different molar ratios. Each experiment has been performed in three replicates. *Red and blue lines* represent the percent decrease in absorbance upon incubation with apo- and holo-SCGN at different molar ratios, respectively. SCGN, secretagogin.

The stress-inducible expression of SCGN points toward a possible chaperone-like activity of SCGN. To establish this, we investigated if SCGN would act as an antiaggregant against various model proteins: alcohol dehydrogenase (ADH), citrate synthase (CS), lysozyme, and malate dehydrogenase (MDH). While the aggregation of the heat-labile enzymes ADH, CS, and MDH was induced by heating them at 41.5, 45, and 50 °C respectively, the aggregation of lysozyme was induced chemically by incubation with 20 mM DTT (30, 31, 32, 33). These are standard conditions to yield maximum aggregation with given substrates and are described in Methods. The aggregation kinetics of the substrates was monitored either alone or in the presence of BSA (control) or SCGN. Since SCGN is a Ca^2+^ sensor protein, all the experiments described above were performed either in the presence of EDTA or Ca^2+^. As expected, the sample turbidity of the substrates alone (monitored at 465 nm, 500 nm, 450 nm, and 360 nm for ADH, CS, MDH, and lysozyme, respectively) rapidly increases with time and eventually reaches a maximum saturation represented as a plateau phase (Fig. 2, *A*–*H*). BSA, used as a control, was merely effective or even displayed slightly enhanced aggregation (with ADH and MDH) but did not reduce aggregation, which is consistent with earlier reports (34). However, the incubation of substrates with SCGN significantly decreases aggregation, more effectively in the presence of Ca^2+^ (Fig. 2, *A*–*D*). In the presence of Ca^2+^, the aggregation of substrates was suppressed substantially as compared to that observed in the absence of Ca^2+^ (Fig. 2, *E*–*H*). While SCGN could reduce the aggregation in the absence of Ca^2+^, the efficacy of chaperone action of SCGN was increased significantly in the presence of Ca^2+^ (80–90%) (Fig. 2,*I*–*L*). In the case of ADH, about 50% protection was observed in the presence of EDTA, which increased to 90% in the presence of Ca^2+^ (Fig. 2*I*). At a 1:2 M ratio of SCGN and substrates, SCGN could reduce scattering (absorbance value) to ∼40%, while at a ratio of 2:1, the inhibition of aggregation was more than 80%, suggesting dose-dependent inhibition of aggregation by SCGN (Fig. 2,*I*–*L*). These results suggest that Ca^2+^ drastically increases the anti-aggregation activity of SCGN and might be a prerequisite for efficient chaperone action of SCGN in the cellular milieu.

### SCGN stabilizes luciferase activity against heat shock

**Figure 3:**
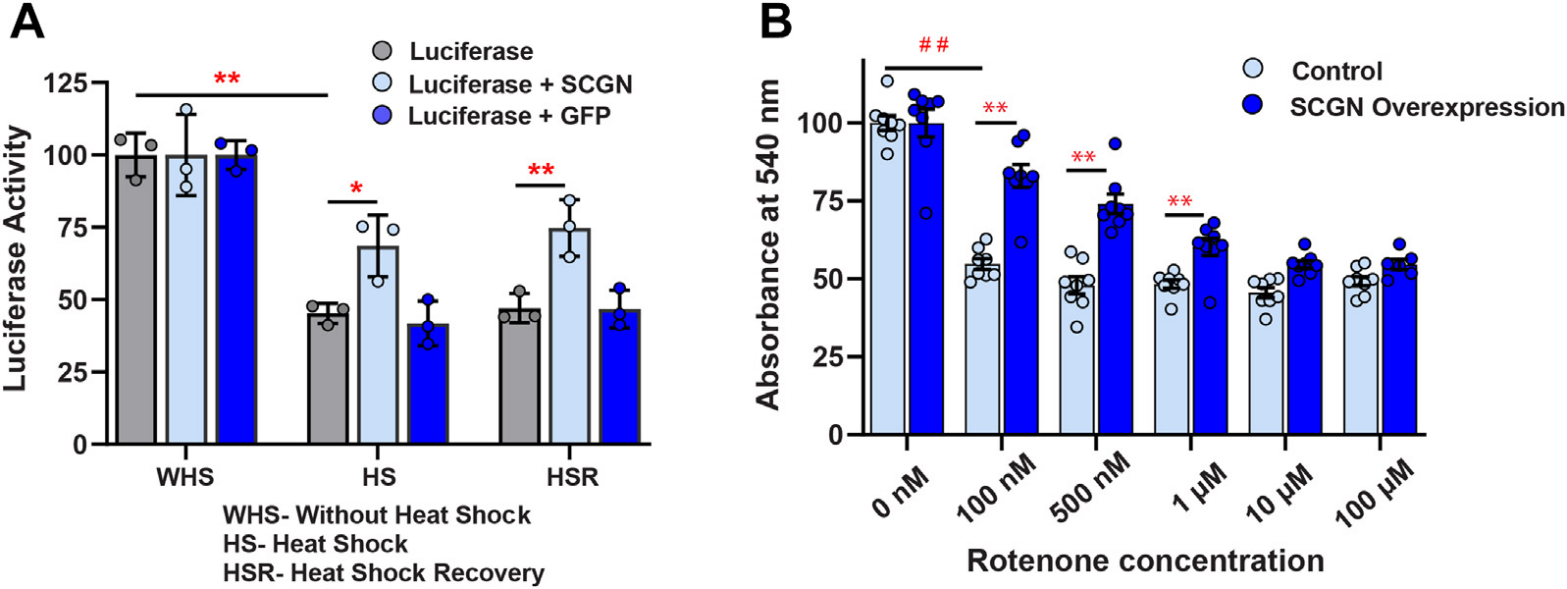
**SCGN overexpression rescues heat shock–induced misfolding of luciferase and prevents cell death against oxidative stress.***A*, percent luciferase activity of control or SCGN-overexpressing HEK-293T cells treated with heat shock (HS), without heat shock (WHS), and after heat shock recovery for 4 h (HSR). Untransfected cells and GFP transfected cells were kept as control. ∗ Represents *p*-value <0.05 and ∗∗ represents *p*-value <0.01 with a minimum of three biological replicates in a group. *B*, MTT assay of rotenone-treated HEK-293T cells with or without SCGN overexpression incubated with 100 nM, 500 nM, 1 μM, 10 μM, and 100 μM rotenone for 24 h. Cell viability was measured as a measure of absorbance at 540 nm. ∗ Represents *p*-value <0.05 and ∗∗ represents *p*-value <0.01 with a minimum of eight biological replicates in a group. SCGN, secretagogin. ## denotes significance of difference between control and 100 nM rotenone treatments, *p*-value <0.01.

Having established that SCGN possesses strong anti-aggregation properties toward general protein substrates, we explored the chaperone activity of SCGN in the cellular milieu by luciferase assay. Taking advantage of the fact that luciferase loses its activity when exposed to heat shock, we checked if SCGN overexpression would rescue luciferase from heat-induced loss of activity. HEK-293T cells were cotransfected with a pGL3 vector containing luciferase construct and eGFP(N3)-SCGN construct. SCGN overexpression was validated in HEK-293T cell line before performing the experiment using RT-PCR and Western blot (Fig. S1). To rule out the possibility of GFP demonstrating any activity, cells transfected with eGFP(N3) plasmid that express GFP alone were used as controls. The expression of other heat-inducible chaperones was suppressed by pretreating the cells with cycloheximide. Under our experimental conditions of heat shock in luciferase-transfected HEK-293T cells (control), we observed about 60% loss of luciferase activity, whereas in SCGN-overexpressing cells, the loss of luciferase activity was ∼35% (Fig. 3*A*). Moreover, SCGN overexpression further increased luciferase activity up to 80% after 4 h of recovery, suggesting the role of SCGN in refolding and regaining luciferase activity. The control and GFP-transfected cells did not show any increase in luciferase activity.

### SCGN protects cells during oxidative stress

Under stress, the expression of HSPs is increased to alleviate protein misfolding and cell death. As SCGN expression was temporally altered by heat and oxidative stress and SCGN exhibits chaperone activity both *in vitro* and *ex vivo*, we assessed if SCGN action is similar to that of an HSP aiding cell survival during cellular stress. Cell death was induced by treating HEK-293T cells with increasing concentrations of rotenone. Cell viability was examined by MTT assay in the rotenone-treated cells with or without SCGN overexpression (Fig. 3). A substantial decrease (up to 40%) in cell viability was observed with increasing concentrations of rotenone (100 nM, 500 nM, 1 μM, 10 μM, and 100 μM) (Fig. 3*B*). In contrast, SCGN overexpressing cells were up to 35% more viable than the control cells, demonstrating that SCGN plays a role in rescuing from cell death.

### Closed to an open conformational transition of SCGN by Ca^2+^

Since SCGN is a Ca^2+^ sensor protein that exhibits Ca^2+^-dependent molecular chaperone activity, we next analyzed the effect of Ca^2+^ on the structural rearrangement of SCGN using small-angle X-ray scattering (SAXS). To ensure sample purity and homogeneity, a quality check of purified samples was performed before SAXS experiments by SDS-PAGE and gel filtration analysis (Fig. S2, *A* and *B*). The homodispersity of the SCGN samples was also ensured by dynamic light scattering analyses prior to SAXS (Fig. S2*C*). SAXS data of protein solutions in the range of 5 to 17 mg/ml concentrations and of the respective matched buffers as blank controls were collected. Data obtained with increasing concentrations of SCGN exhibit a profile characteristic of a compact globular molecule as depicted from the log I(q) *versus* log q plot (Fig. S3*A*). The Kratky plot confirms a well-folded conformation and exhibits only subtle differences between apo, Ca^2+^-, and Mg^2+^-bound SCGN (Fig. S3*B*). At low q values, a plot of ln(I(q)) *versus* q^2^ is linear and independent of protein concentration indicating the homogeneity of the protein sample as observed in the Guinier plot (Fig. S4*A*). The Guinier R_g_ calculated from the Guinier plot correlates well with distance distribution function (P(r)) R_g_ and CRYSOL R_g_ calculated from the crystal structures (PDB ID: 2be4 and 6jlh) of *Danio rerio* SCGN (Fig. S4*B*). The protein samples remained unaffected post-X-ray exposure as confirmed with SDS-PAGE analyses of post-SAXS samples (Fig. S5*A*). This was further corroborated with MALDI analysis of SCGN samples before and after X-ray exposure. The m/z peak corresponding to a molecular weight of 33 kDa (monomeric SCGN) did not alter (Fig. S5, *B* and *C*).

**Figure 4:**
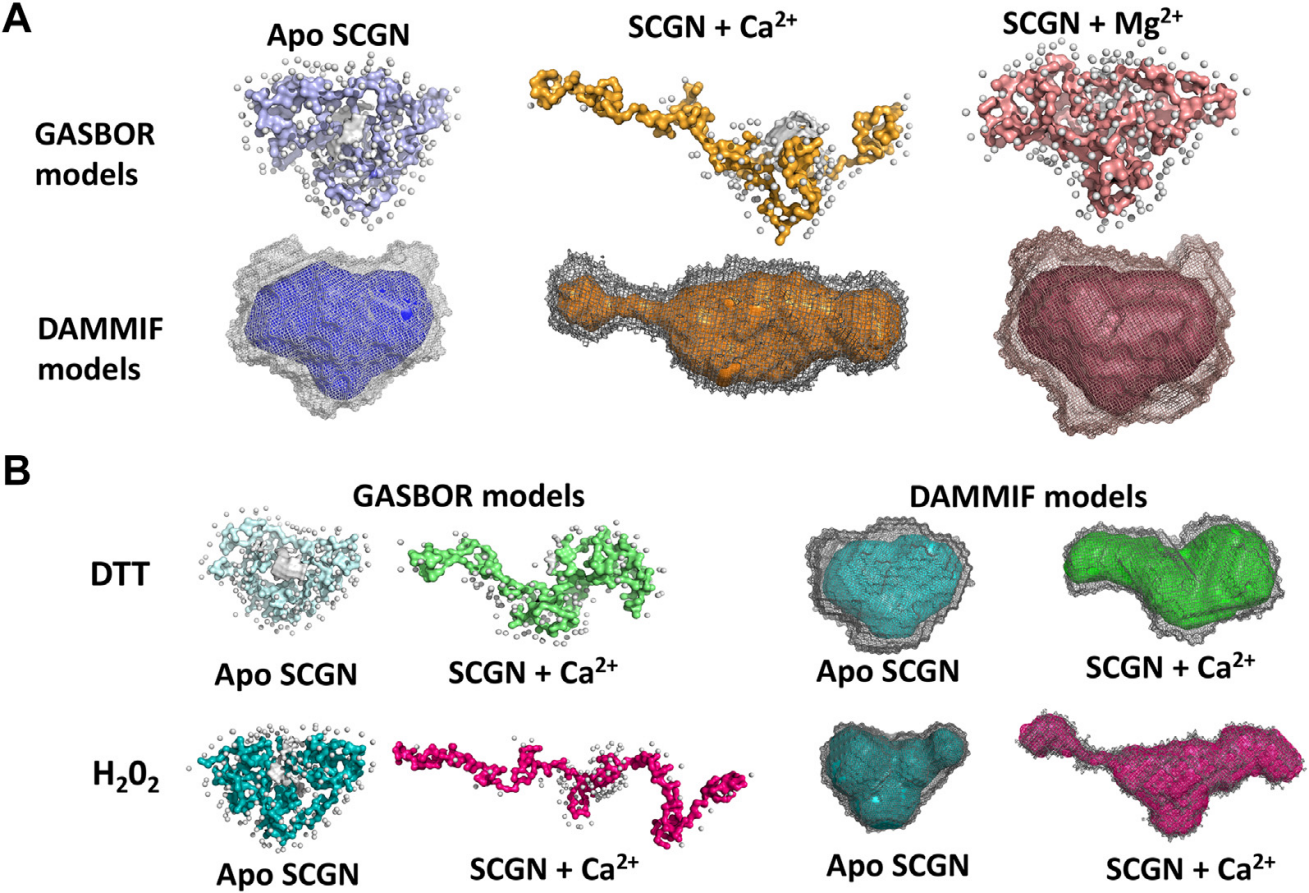
**Ca**^**2+**^**binding leads to elongation of SCGN**. SAXS-derived GASBOR (*right*) and DAMMIF *ab initio* models (*left*) of SCGN. The SAXS data were collected with ∼10 mg/ml SCGN for (*A*) Apo, Ca^2+^- (8 mM) and Mg^2+^- (8 mM) bound SCGN, and (*B*) SCGN in reducing (5 mM DTT) and oxidizing conditions (100 μM H_2_0_2_). SAXS, small-angle X-ray scattering; SCGN, secretagogin.

At protein concentrations of ∼5 mg/ml, the radius of gyration (R_g_) of apo-protein was found to be 23.4 Å, which increases drastically to 37.7 Å upon the addition of Ca^2+^. Interestingly, at a higher protein concentration (13.5 mg/ml), the radius of gyration in the presence of Ca^2+^ increased to 47.5 Å. On the other hand, even the saturated level of Mg^2+^ (8 mM) did not induce any change in R_g_ value (23.7 Å) with increasing concentrations, suggesting a Ca^2+^-specific structural rearrangement (Table S1). Structural models prepared using *ab initio* programs GASBOR and DAMMIF suggest that both the apo- and Mg^2+^-bound forms of SCGN exist as a V-shaped globular molecule. This conformation is transformed to an open, elongated structure by Ca^2+^, thus forming a cleft plausibly for substrate binding (Fig. 4*A*).

To rule out the possibility of the Ca^2+^-dependent increase in R_g_ value being a result of dimerization or higher oligomer formation, we recorded structural changes in SCGN in reducing and oxidizing milieu using SAXS analysis. In reducing conditions (in the presence of 5 mM DTT), the R_g_ value of apo-SCGN was 22.8 Å, which was slightly increased to 25.6 Å in oxidizing conditions (in the presence of 100 μM H_2_O_2_). SCGN exists as a monomer in reducing conditions, while in oxidizing conditions, the protein tends to oligomerize. However, the effect of Ca^2+^ was more pronounced in oxidizing conditions. At low protein concentrations of holo-SCGN under oxidizing conditions, the R_g_ value was increased to 37.4 Å, and at higher protein concentrations, the R_g_ value was further increased to 46.9 Å. However, the R_g_ value of holo-SCGN in reducing conditions was increased only up to 33.3 Å, suggesting that the increase of R_g_ is not due to disulfide-mediated dimerization but is a consequence of Ca^2+^ binding (Table 1). This further confirms that dimerization of the Ca^2+^-bound form in oxidizing conditions is accentuated at higher protein concentrations, as reported earlier (24, 35).

**Table 1:** SAXS-derived structural parameters of SCGN at various concentrations in oxidizing (100 μM H_2_O_2_) and reducing conditions (5 mM DTT) in the presence and absence of Ca^2+^ using ATSAS software analysis

SCGN (mg/ml)		I_0_/c	R_g_^Guinier^ (Å)		R_g_^P(r)^ (Å)	D_max_ (Å)	Guinier range		MW porod (kDa)
DTT	8.7	0.32	22.8	± 1.2	23.0	75	11	62	32.3
	11.51	0.32	22.9	± 1.0	22.9	74	12	61	31.9
	16.65	0.31	21.5	± 1.7	22.0	67	7	66	31.6
DTT + Ca^2+^	8.75	0.39	28.8	± 1.0	28.6	92	15	47	35.3
	11.34	0.4	29.2	± 0.9	28.9	95	11	45	34.9
	18.5	0.55	33.3	± 2.9	37.5	167	15	40	44.1
H_2_O_2_	5.39	0.43	25.6	± 0.6	26.6	106	9	54	34.6
	9.18	0.4	24.2	± 1.1	23.9	76	6	57	32.1
	10.56	0.38	23.5	± 0.4	23.4	76	11	59	32
H_2_O_2_ + Ca^2+^	4.9	0.69	37.4	± 1.3	41.6	184	4	29	50.7
	7.34	0.64	35.5	± 1.5	40.8	177	10	37	51.7
	15.3	0.95	46.9	± 2.8	52.5	216	8	26	84.5

It is also important to note that the D_max_ for Ca^2+^-bound protein samples (∼8 mg/ml) in reducing conditions is increased to 92 Å (from 75 Å for apo-SCGN), while in oxidizing conditions, D_max_ increased considerably to 184 Å (from 106 Å for apo-SCGN). This supports our earlier data which showed that SCGN forms oligomers in a concentration-dependent manner under oxidizing conditions, while the addition of DTT abrogates oligomerization (24, 36). While the GASBOR and DAMMIF models for apo SCGN with DTT and H_2_O_2_ are fairly similar, the model of holo-SCGN in oxidizing condition appears more elongated, in accordance with increased R_g_ values (Fig. 4*B*). On the other hand, Ca^2+^-bound SCGN in the presence of DTT remains a monomer that adopts a partially open conformation with the two terminal domains flanking away from the central region. Thus, intracellular Ca^2+^ concentration and cellular redox status would enable SCGN to adopt multiple conformations resulting in diverse substrate binding, a characteristic of a molecular chaperone.

### Structural and mechanistic insights into Ca^2+^-dependent enhancement of chaperone action of SCGN

**Figure 5:**
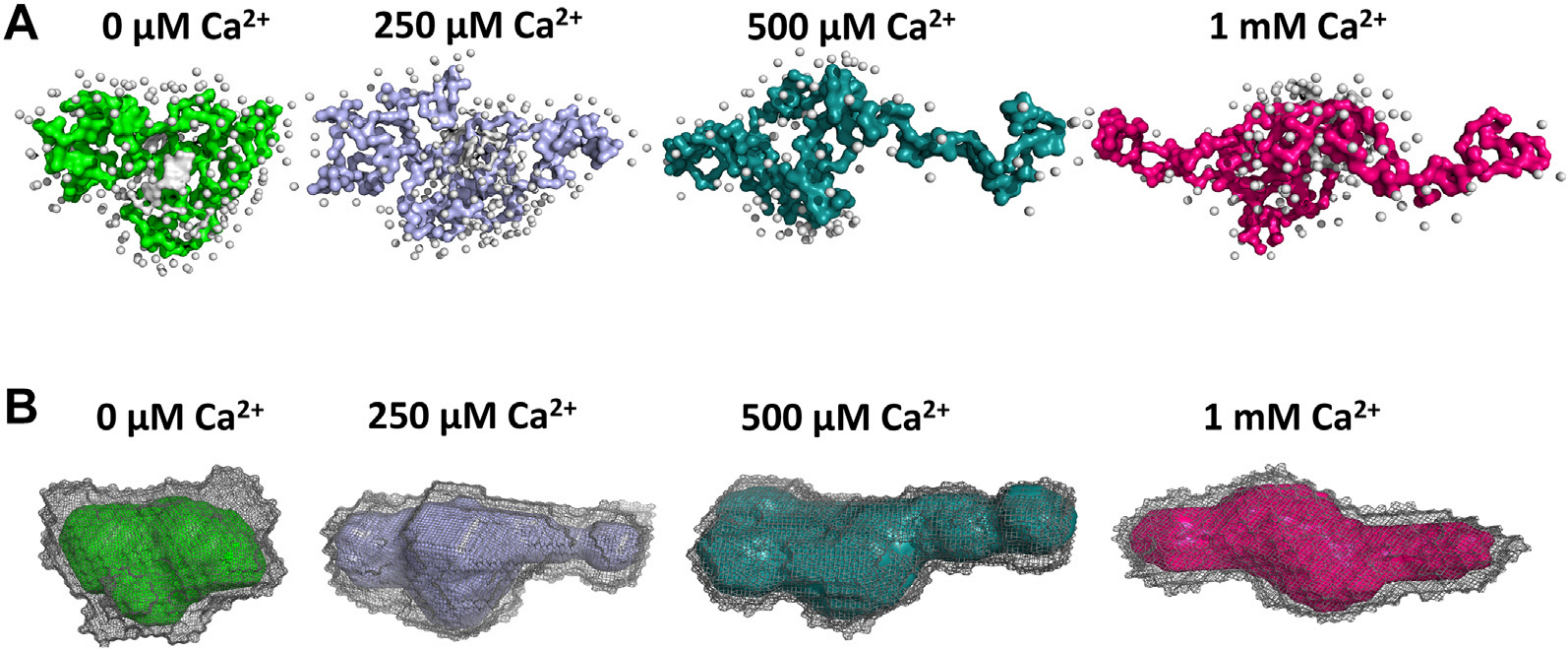
**Closed to open conformational transition of SCGN by Ca**^**2+**^**as shown by SAXS modeling analysis**. *A*, the GASBOR *ab initio* model and (*B*) DAMMIF molecular models of SCGN constructed upon Ca^2+^ titrations ranging from 50 μM to 1 mM. The BioSAXS data of SCGN were recorded at 7 mg/ml concentration. SAXS, small-angle X-ray scattering; SCGN, secretagogin.

Having identified the closed (apo-SCGN) and open (holo-SCGN) conformations of SCGN, we decided to dissect the dynamics of these conformational changes. As shown above, Ca^2+^ binding causes major rearrangements with the relocation of both terminal domains and the formation of an open elongated structure, which is largely an extended arrangement of the domains. To reveal the progression of intermediate events, structural changes in SCGN (7 mg/ml) were monitored by SAXS by the sequential addition of Ca^2+^. The R_g_ value of the apo-SCGN (23.2 Å) did not change significantly when titrated with Ca^2+^ up to 100 μM Ca^2+^ but reduced marginally to 22.7 Å at 150 μM Ca^2+^ (Table 2). At 200 μM Ca^2+^ concentration, the R_g_ and D_max_ values were restored to that of the apo-protein. At 250 μM Ca^2+^, an expansion of both R_g_ (26.8 Å) and D_max_ (93 Å) was noted, signaling the initiation of a conformational transition. At this Ca^2+^ concentration, the V-shape geometry of apo-SCGN turns toward a U-shape, as seen in the GASBOR model, whereas one of the terminal domains is already flanked away as seen in the DAMMIF model (Figs. 5, and S7). At intermediate Ca^2+^ concentrations (250 μM [Ca^2+^] for DAMMIF model and 400 μM [Ca^2+^] for GASBOR model), the protein begins to open up, as displayed by the movement of one domain being pushed away from the center of the protein. Finally, at saturated Ca^2+^ concentrations (1 mM Ca^2+^), the R_g_ (31 Å) and D_max_ (123 Å) values increased, indicating the opening of the molecule into an extended conformation (Table 2). In other words, the sequential addition of Ca^2+^ induces a structural rearrangement that initiates the movement of the N- and C-domains away from the middle domain, creating a crevice in the central domain leading to the creation of a site ready and available for substrate binding. The global structural changes induced by Ca^2+^ display step by step transformation of SCGN into a molecular chaperone.

**Table 2:** R_g_ values obtained from the Guinier approximation of the SAXS intensity profiles

Ratio Ca^2+^/SCGN	[Ca^2+^]	I_0_/c	R_g_^Guinier^ (Å)		R_g_^P(r)^ (Å)	D_max_ (Å)	Guinier range		MW Porod (kDa)
-	0 μM	0.32	22.8	± 1.2	23.0	75	11	62	32.3
0.23	50 μM	0.3	23.2	± 0.3	23.7	79	17	60	32.8
0.46	100 μM	0.31	23.4	± 0.3	23.6	79	7	60	31.7
0.69	150 μM	0.27	22.7	± 0.2	22.8	73	8	62	32
0.92	200 μM	0.32	23.3	± 0.2	23.3	77	8	60	32.6
1.15	250 μM	0.38	26.8	± 0.3	27	93	13	51	34.6
1.38	300 μM	0.37	28	± 2.2	27.2	86	9	45	34.7
1.84	400 μM	0.4	29.2	± 0.6	31.6	132	7	46	39.1
2.3	500 μM	0.44	30.1	± 1.1	31.2	107	10	45	38.9
4.6	1 mM	0.39	31.2	± 0.7	32.6	123	5	43	37.1

SCGN (7 mg/ml) was titrated with increasing Ca^2+^ concentration ranging from 50 μM to 1 mM.

**Figure 6:**
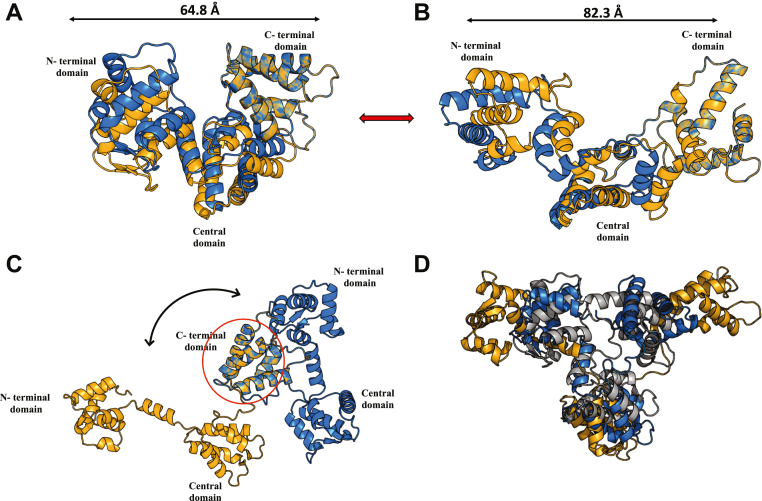
**Structural analysis of apo to holo transitional states of SCGN.** Multi-FOXS models showing best-fit ensembles of (*A*) apo and (*B*) Ca^2+^-bound SCGN. The overlay of two randomly selected conformational ensembles is represented in *blue* and *orange*. *C*, Apo (*blue*) and Ca^2+^-bound SCGN (*orange*) models overlap only in the C-terminal domain, while the N-terminal domain completely shifts by 180°. *D*, superimposition of three randomly selected ensembles of SCGN at 250 μM Ca^2+^ (transition state) (represented in *blue*, *gray*, and *orange*). They do not overlap demonstrating flexible states of mixed ensembles. SCGN, secretagogin.

To understand the multiple states of SCGN conformation during Ca^2+^ binding, we analyzed the SAXS data using the MULTI-FOXS program, which provides population-weighted ensembles fitting to a SAXS profile of the protein in solution (37). Ensembles of models obtained were based on the crystal structure of apo-SCGN (PDB ID: 2be4 (38)) and Ca^2+^-bound SCGN (PDB ID: 6jlh (3)) from *D. rerio*. The MULTI-FOXS analysis reveals that the SAXS-based ensembles of the apo-state of mouse SCGN correlate well with the crystal structure of *D. rerio* apo-SCGN (PDB ID: 2be4) (Fig. 6*A*). The model built from the SAXS data of the SCGN at saturated Ca^2+^ concentration fits well with the crystal structure of Ca^2+^-bound *D. rerio* SCGN (PDB ID: 6jlh) (Fig. 6*B*). A substantial expansion of SCGN molecule from about 65 Å in the apo form to 82 Å in Ca^2+^-bound form is observed indicating the adoption of an open conformation exerted by Ca^2+^ binding to the molecule (Fig. 6, *A* and *B*). The models of apo- and Ca^2+^-bound SCGN overlap only in the C-terminal domain, whereas Ca^2+^ causes a large shift of the N-terminal domain along with the central region (Fig. 6*C*). The intermediate state during this transition captured at 250 μM [Ca^2+^] is a mixed ensemble of three randomly selected ensembles with several different orientations that do not overlap at all, demonstrating the high flexibility of this intermediate state (Fig. 6*D*). This is further corroborated by a sharp increase in the R_g_ value (Fig. 6*D* and Table 2). A structural comparison of the SAXS-derived model of Ca^2+^-bound SCGN with the Ca^2+^-bound crystal structure of a related Ca^2+^ sensor, calbindin-D28K, highlights the distinctive structural transformation of SCGN (Fig. S8). Unlike SCGN, Ca^2+^ does not induce a drastic conformational change in calbindin D28K (Fig. S8). We thus demonstrate how Ca^2+^ may activate SCGN by transforming the molecule from closed to open conformation to be geared for performing Ca^2+^-dependent functions.

### Ca^2+^ enables the binding of client proteins to SCGN

**Figure 7:**
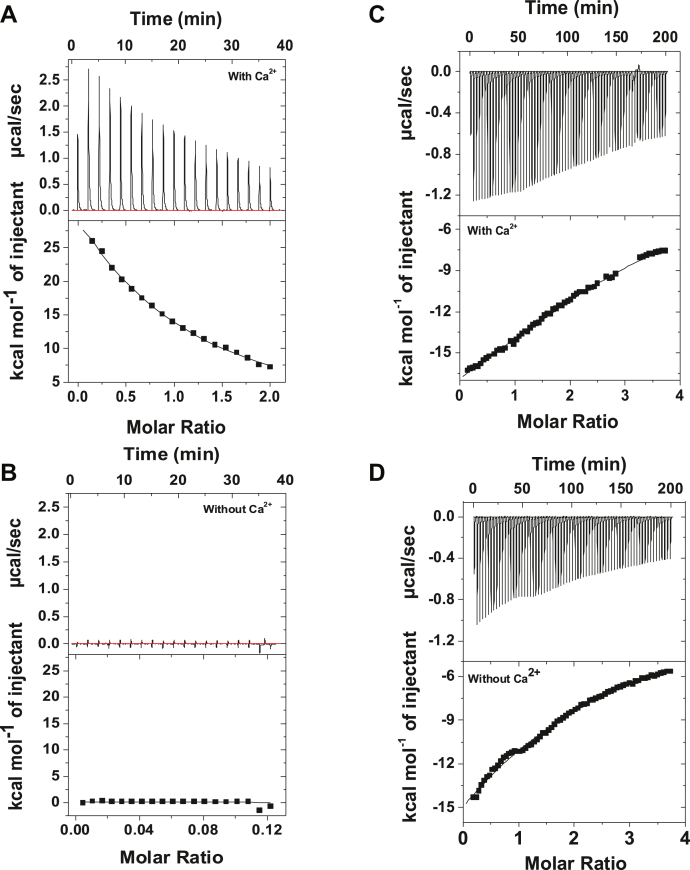
**Client proteins binding to SCGN.***A* and *B*, ITC thermogram depicting heat change per injection upon titration of α-synuclein (600 μM) with SCGN (60 μM): (*A*) in the presence of 2 mM Ca^2+^ and (*B*) in the absence of Ca^2+^. *C* and *D*, ITC thermogram of SCGN-insulin binding. ITC experiments were carried out with SCGN (30 μM) and research grade recombinant insulin (1 mM) (*C*) in the presence of 3 mM Ca^2+^, and (*D*) absence of Ca^2+^. Appropriate blanks were subtracted from the data. All the experiments were repeated at least thrice. The presented thermograms are from the representative set. ITC, isothermal titration calorimetry; SCGN, secretagogin.

It is seen above that Ca^2+^ transforms SCGN from closed to open conformation. To investigate how this process impacts the interaction of SCGN with client proteins, we selected α-synuclein, an intrinsically unstructured protein prone to aggregation and implicated in proteinopathy, and insulin known to interact with SCGN (6, 23). The binding of α-synuclein to SCGN was examined by isothermal titration calorimetry (ITC). The thermogram of SCGN binding to α-synuclein in the presence of Ca^2+^ follows an endothermic process and data best fit to one-set of binding site model (Fig. 7*A*). The binding of SCGN to α-synuclein is an enthalpically unfavorable but entropically favorable process with enthalpy change (ΔH) being 1.24 cal/mol and entropy change (ΔS) being 435 cal/mol/deg. The dissociation constant (K_d_) for binding of α-synuclein to SCGN is 110 μM, indicating a weak binding. We further confirmed it by monitoring the stability of SCGN–α-synuclein complex using analytical gel filtration. We found that SCGN does not form a stable complex with α-synuclein suggesting a weak interaction with the substrate (Fig. S9). The interaction of insulin with SCGN was also studied by ITC. As per analysis, the Ca^2+^-free SCGN binds insulin with an affinity of (K_d_) ∼213 μM which, in the presence of Ca^2+^, increases up to ∼73 μM (Fig. 7, *C* and *D*). These results demonstrate that Ca^2+^ modulates the binding positively. Our results align with the finding that the binding of target proteins to chaperones is weak which enables target proteins to dissociate easily from the complex to be available to form stronger interactions of the folded state (39). On the other hand, in the absence of Ca^2+^, we did not observe any measurable heat change (Fig. 7*B*), suggesting that binding of SCGN and α-synuclein takes place only when Ca^2+^ is present. This highlights the indispensable role of Ca^2+^ in mediating structural changes in SCGN, which allows substrate binding.

### The substrate selectivity for chaperone action is determined by the distinct domains of SCGN

**Figure 8:**
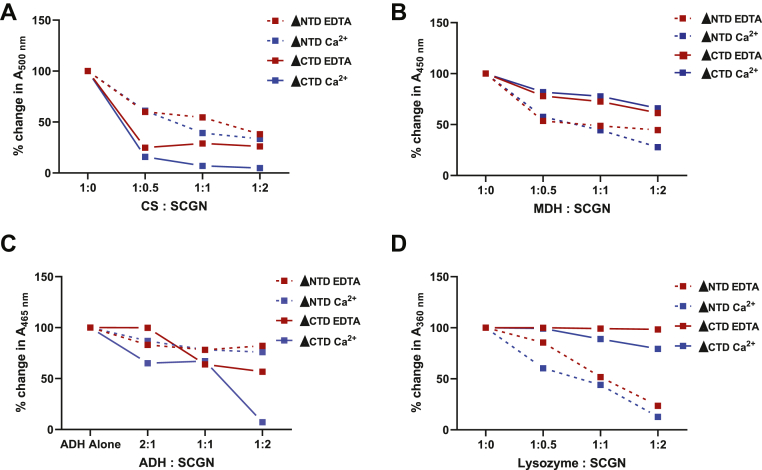
**Central domain of SCGN is indispensable for chaperone activity.** Aggregation kinetics of model substrates: (*A*) CS, (*B*) MDH, (*C*) ADH, and (*D*) lysozyme was monitored in the presence of varying concentrations of ΔNTD-SCGN and ΔCTD-SCGN in the presence of either 10 μM EDTA (*red*) or 2 mM Ca^2+^ (*blue*). The percentage decrease in absorbance was calculated and plotted at the saturation point. ADH, alcohol dehydrogenase, CS, citrate synthase; MDH, malate dehydrogenase; SCGN, secretagogin; ΔCTD, C-terminal deleted; ΔNTD, N-terminal deleted.

The movement of the terminal domains by Ca^2+^ leading to the creation of a binding site for substrates led us to assign the role of each domain, if any, in imparting chaperone activity. To this end, we prepared two deletion constructs of SCGN: C-terminal deleted (ΔCTD) and N-terminal deleted (ΔNTD) constructs. The recombinant ΔCTD (20.4 kDa) and ΔNTD (19.1 kDa) proteins having the central domain in common were tested for chaperone activity. The choice of selection of two domains out of the three was to have stable protein preparations. While both proteins possess chaperone activity, there was a variation in the ability of ΔCTD (20.4 kDa) and ΔNTD (19.1 kDa) to suppress aggregation at identical protein to substrate ratios, depending on the model substrate. For example, in the case of CS, ΔCTD was more efficient than ΔNTD in precluding aggregation, while in the case of lysozyme, ADH, and MDH, ΔNTD was more efficient (Fig. 8). Apart from these results, our previous studies also displayed variations in the efficiency of suppressing aggregation. In the case of insulin, ΔCTD efficiently halts its aggregation (5), while for α-synuclein, ΔNTD of SCGN is more effective in preventing fibrillation (23). We speculate that the size, shape, and charge of the substrate play an important role in deciding its interaction with either the N-terminal or C-terminal domain upon binding at the central domain. Thus, distinct regions of SCGN play a role in deciding how it interacts with its substrate for chaperone action.

## Discussion

Dysregulation of SCGN homeostasis is implicated in the progression of several pathophysiologies like diabetes, neurodegeneration, and cancer (4, 7, 9, 10, 11, 12, 13, 18). A common causative factor in the onset and advancement of these disorders is cellular stress. There can be various factors that are responsible for cellular stress, namely heat shock, redox imbalance resulting in oxidative stress, heavy metal exposure, and deregulation of Ca^2+^ homeostasis (40, 41, 42, 43, 44). Multiple studies have reported a change in serum/plasma levels of SCGN during diabetes, neurodegeneration, and cancer, suggesting it to be a potential biomarker for these diseases (4, 9, 11, 45). Further, overexpression of SCGN has been shown to rescue neurodegeneration and insulin resistance (5, 7). SCGN expression is known to be regulated by the activation of Ca^2+^ permeable TRPV1 channels (46), and TRPV1 agonists also upregulate *Hsp70* (47, 48) that suggests a relation between SCGN expression and heat shock response. During stress, the transcript and protein expression dynamics of most of the stress proteins is inconsistent (49, 50). Upon cellular stress, the mRNAs of stress proteins exhibit transient spike and decline, while their protein levels either elevate and maintain a new steady-state (GRP78 and HSP90B1) or remain constant throughout response (HSP90AA1) (49, 50). The *Scgn* transcript dynamics concurs with those of a few stress-responsive proteins like GRP78, HSP90B1, and P58IPK (49). The increased *Scgn* transcript levels during the early response, however, do not correlate with the increase in intracellular protein levels. Further, during the late response to oxidative stress, SCGN protein is maintained at a lower steady-state level, unlike that of constitutively-expressed GRP78 or HSP90B1 chaperones (49). We speculate that this could be either due to yet unknown posttranscriptional regulation or due to secretion of SCGN into the media poststress. Despite these intriguing correlations, the precise role and regulation of SCGN expression during cellular stress remain unknown.

We further demonstrate that SCGN effectively prevents both the heat-inducible as well as the chemically inducible (DTT) aggregation of a wide range of proteins *in vitro* in a Ca^2+^-dependent manner. This finding, along with the interaction of SCGN with several *bona fide* molecular chaperones and 26S proteasome subunits, indicates that SCGN could be a part of the protein folding assembly to aid in the protein homeostasis and/or folding process (22, 51). Another layer of regulation of SCGN activity could stem from the localization of SCGN and the corresponding organelle milieu that dictates the Ca^2+^ and the redox signal/stimulus needed to activate SCGN. We have reported earlier that in an oxidizing environment (like in ER), SCGN could act as a Ca^2+^ buffer, while in the cytosol’s reducing environment, it might act as a Ca^2+^ sensor (6, 35, 36, 52). Thus, we speculate that SCGN would largely function as a chaperone in the ER’s oxidizing environment with high Ca^2+^ concentration. However, in the cytosol, an increase in cytosolic Ca^2+^ concentration upon release from intracellular stores could lead to the activation of SCGN.

We further checked if SCGN could mediate its chaperone functions in a cellular scenario. We found that SCGN overexpression prevents the loss of luciferase activity in HEK-293T cells. Our results further bolster the earlier published results of SCGN-mediated inhibition of fibrillogenesis of insulin and α-synuclein (5, 23). Based on these results, it is reasonable to anticipate that SCGN would indeed protect other aggregation-prone proteins, specifically those implicated in neurodegenerative diseases, such as Aβ-42 and Tau from undergoing fibrillogenesis. We also demonstrated that SCGN overexpression could protect cells from oxidative stress. This is in line with the earlier reports that demonstrated a correlation of SCGN expression with ER stress, protein folding, quality control, and cell survival (46, 53). This is possibly mediated by maintaining the level and activity of proteins, such as ubiquitin carboxyl-terminal hydrolases USP9X and USP7 that boost β-cell proliferation, implying a direct role in protein folding (5).

We now identify chaperone activity as one of the crucial functions of SCGN vis-a-vis Ca^2+^ and elucidate why deregulated SCGN levels accelerate protein misfolding disorders. Reduced levels of SCGN in certain tissues, as noted in protein folding diseases (7, 9, 24), and increased release of SCGN in response to endoplasmic reticulum stress (4) is consistent with our findings. We provide a rationale for a direct interplay among the three: (i) cellular stress, (ii) deregulation of Ca^2+^ and/or SCGN expression levels, and (iii) protein misfolding diseases, such as Parkinson’s and Alzheimer’s disease. A reduction in SCGN expression, or even a perturbation in Ca^2+^ homeostasis, would consequently affect SCGN’s propensity to function as a chaperone, which would hasten the aggregation of the concerned proteins, accelerating the onset of disease.

We next checked the alterations in structural features of SCGN by Ca^2+^ in the augmentation of SCGN’s chaperone activity. BioSAXS has provided us with an innovative perspective on the Ca^2+^-dependent folding mechanism of SCGN in unprecedented detail. The pair distribution curve (the P(r) function), the Guinier plot, I_o_/C value, and concentration-dependent monitoring of R_g_ values are good quality control checks, and the current work appropriately accounted for them. Monitoring the ensembles of structures during the sequential addition of Ca^2+^ has allowed us to display the presence of a conformational switch of SCGN. Ca^2+^ binding triggers the drifting of both terminal domains away from the central domain, creating an open groove, enough to accommodate the binding of a broad range of substrates. The ends of both terminal domains appear to move away from each other, with the connecting residues—between NTD and the central domain (residues 94–100) and between the central domain and CTD (residues 173–178)—acting like flexible hinges (computed using the HingeProt program) (54). This drifting movement of both terminal domains serves as a mechanistic model for the transition from the closed (apo form) to open and activated conformation (Ca^2+^ bound). It is to be noted that a similar Ca^2+^-dependent elongation of the structure is reported in gelsolin, a Ca^2+^-binding protein (55), which is not known to act as a chaperone. Calbindin D28K is another hexa EF-hand protein that does not show such structural change upon Ca^2+^ binding (56). Thus, our study opens a new avenue for monitoring structural transitions in Ca^2+^-dependent chaperones to better understand their mechanism of action.

To summarize, the present study describes a previously unidentified function of SCGN wherein it not only efficiently chaperones various protein substrates but also helps in maintaining a healthy proteome in cells during conditions of stress. The interplay between stress, neurodegenerative diseases, and SCGN expression was unclear for a long time. The importance of SCGN as a Ca^2+^-dependent chaperone and its role in stress physiology has helped resolve this enigma. This study enables the extraction of important insights into the multifunctional nature of SCGN and lays a foundation for future therapeutic discoveries for neurodegenerative diseases exploiting the SCGN model.

## Experimental procedures

### Chemicals and antibodies

Alcohol dehydrogenase lyophilized powder sourced from baker’s yeast was obtained from SRL. CS and MDH sourced from the porcine heart (Sigma), and lysozyme from chicken egg white (Sigma) were used for chaperone assays. The antibodies used in these experiments are anti-SCGN bs-(11744R; Bioss), anti-Hsp70 (MA3-007; Invitrogen), anti-GRP78(C50B12; CST), anti-β-Actin (bs0061R; Bioss), and HRP conjugated secondary antibody (bs0295G; Bioss).

### Cloning and protein purification

The overexpression of mouse SCGN cloned in pET21b expression vector in *E. coli* BL21-DE3 cells was induced with 1 mM IPTG at *A*_600_. of 0.6 and incubated at 25 °C for 10 h postinduction as reported previously (36, 57). Briefly, the soluble fraction of the bacterial pellet was loaded on a Ni-NTA-Sepharose (GE Healthcare) column, which was pre-equilibrated with 50 mM Tris, pH 7.5, 100 mM KCl (equilibration buffer), and washed with wash buffer (50 mM Tris, pH 7.5, 100 mM KCl, and 2% Triton X-100) followed by a wash with equilibration buffer. The protein was eluted with a gradient of 0 to 250 mM imidazole in 50 mM Tris, pH 7.5, and 100 mM KCl buffer.

### *Δ*NTD-SCGN and ΔCTD-SCGN

The overexpression of ΔNTD-SCGN or ΔCTD-SCGN in *E. coli* BL21-DE3 cultures was induced with 1 mM IPTG at 37 °C after the *A*_600_ reached about 0.6 and incubated at 18 °C for 16 h postinduction (23). The ΔNTD-SCGN was purified by employing a two-step procedure. The soluble fraction from the bacterial pellet was first loaded onto a phenyl-Sepharose column (GE Healthcare). After one wash with lysis buffer, the protein was eluted in an elution buffer of 50 mM Tris, pH 7.5 and 100 µM EDTA. In the second step of purification, the protein eluted in the previous step was loaded on a Q-Sepharose column in the same buffer (50 mM Tris, pH 7.5 and 0.5 mM EDTA). The nonspecific proteins were removed first by a wash buffer containing 2% Triton X-100 followed by a wash buffer without Triton X-100. The protein was eluted using a gradient of 200 mM to 1 M NaCl. The purification of ΔCTD-SCGN was performed on an anion exchange chromatography on a Q-Sepharose resin using the same protocol that is described for ΔNTD-SCGN (23). All the proteins were further purified using size-exclusion chromatography on a Sephadex 75pg column coupled to an FPLC machine (Bio-Rad). Purified proteins were decalcified by incubation with 100 μM EDTA followed by buffer exchange against Chelex-purified buffer till the added EDTA is removed.

### Chaperone activity assay

Aggregation kinetics was monitored in time-dependent mode on a Lambda 35 UV spectrophotometer (PerkinElmer) equipped with a water bath or on a Hitachi Fluorescence Spectrophotometer F-7000 connected to a temperature controller (TCC100). Thermal aggregation of ADH (1.3 μM), MDH (1.5 μM), or CS (2 μM) solutions in 50 mM Hepes, 100 mM KCl, pH 7.5, was induced at 41.5 °C, 50 °C, and 45 °C respectively, and monitored at 465 nm, 450 nm, and 500 nm, respectively (30, 31, 32, 33). Chemical aggregation of lysozyme (10 μM) was initiated by the addition of 20 mM DTT in 50 mM Hepes, 100 mM KCl, pH 7.5 buffer. Change in turbidity of ADH, CS, and lysozyme was monitored by measuring the increase/decrease in absorbance units. Aggregation of MDH was monitored by recording 90° scattering at 465 nm on a fluorescence spectrophotometer. The aggregation kinetics of all the substrates was recorded either with 2 mM Ca^2+^ or with 10 μM EDTA. The reaction mixtures of each substrate with or without SCGN (or BSA as a control) were incubated at respective temperatures with constant stirring and monitored at the abovementioned wavelengths. The reaction mixtures of each substrate with the deletion constructs of SCGN (ΔNTD and ΔCTD) were also incubated at temperatures as mentioned above, and absorbance was monitored at 500 nm, 450 nm, 465 nm, and 360 nm wavelengths for CS, MDH, ADH, and lysozyme, respectively. Experiments were performed with varying stoichiometric ratios of SCGN to substrates (1:1, 1:2, and 2:1). All the experiments were performed in triplicates, and the obtained data were analyzed and plotted using Origin Lab (2019b version) and GraphPad Prism 8. The percent change in chaperone activity of SCGN is calculated using,

$$\;\%\;chaperone\;activity\;of\;SCGN=100-\left(\frac{A_{Substrate}-A_{SCGN\;+\;Substrate}}{A_{Substrate}}\right)\text{ ∗ }100\;$$

where A_Substrate_ is the absorbance at a respective wavelength for the substrates used (ADH, MDH, CS, and lysozyme), and A_SCGN + Substrate_ is the highest absorbance value obtained with substrate and SCGN.

### Cell culture

RIN-5F and HEK-293T cells were cultured in RPMI and DMEM media, respectively, supplemented with 10% fetal bovine serum (containing penicillin, streptomycin, and kanamycin) and maintained at 37 °C in a 5% CO_2_ incubator. Luciferase construct in basic pGL3 vector [procured from Promega luciferase assay system kit (Catalog No. E4030)], pEGFP(N3)-SCGN plasmid, and SCGN-eGFPN3 constructs were used for transient overexpression in HEK-293T cells. Transfection of cells was performed with Lipofectamine 3000 reagent (Invitrogen) according to the manufacturer's protocol.

### Heat shock treatment

RIN-5F cells (0.2 million) were seeded in a T-25 flask. After 24 h, the cells were subjected to heat shock at 42 °C for 45 min. Cells without heat shock were used as control. Samples in triplicate were collected at 0, 1, 2, 3, 4, 5, and 6 h of recovery from heat shock. The cells were either lysed in RIPA buffer for Western blotting or in Trizol reagent for qRT-PCR analysis.

### Rotenone treatment

RIN-5F cells were seeded in a 6-well plate at a density of 2 × 10^5^ cells/well. The next day, the cells were incubated with 1 μM rotenone (Sigma), and the samples were collected at 3, 6, 9, 12, 18, and 24 h. Untreated cells were maintained as control. Cells were lysed in RIPA buffer for Western blotting and in Trizol reagent for RNA preparation for qRT-PCR. Each experiment was performed in triplicate.

### Western blotting

Protein in cell lysate was quantified using a Takara BCA protein estimation kit. For each sample, 35 μg total protein was loaded on 12% SDS PAGE gel and transferred onto a nitrocellulose membrane at 100 V for 2 h. After blocking with 5% BSA for 2 h, blots were incubated overnight at 4 °C with either anti-SCGN antibody (1:4000) or anti-HSP70 antibody (1:2000) or anti-GRP78 (1:2000) or β-Actin (1:20,000). The next day, the blots were washed with TBST (50 mM Tris, pH 7.5, 100 mM KCl, 0.1% Tween-20) four times at 10 min intervals. Subsequently, the blots were incubated with anti-mouse or anti-rabbit secondary antibody conjugated with HRP (1:10,000) for an hour. Post incubation, the blots were washed four times every 10 min with TBST and developed using the chemiluminescence method with a Bio-Rad ECL substrate kit. The obtained bands were analyzed using Image J software and normalized with β-actin.

### Quantitative real-time PCR

Total RNA from the heat shock and oxidative stress samples was extracted by Trizol-chloroform method followed by DNase treatment. After assessing RNA quality, 1 μg of total RNA from each sample was used for cDNA synthesis (Takara PrimeScript first strand cDNA synthesis kit). mRNA expression levels were quantified by qRT-PCR on an ABI Prism 7900 HT (Applied Biosystems) using the SYBR Green detection system (Applied Biosystems). The reactions were performed according to the following conditions: initial hold at 95 °C for 30 s, followed by 40 cycles of amplification at 95 °C for 10 s, annealing at 60 °C for 15 s, and extension at 72 °C for 30 s. At least three technical replicates were prepared in each group of samples. β-actin/RPL11 or TBP was used as a reference control. The relative mRNA expression was calculated using the 2^-ΔΔct^ as a mean of at least three technical replicates. Primers for the target genes used for qRT-PCR are listed in Supplementary Information (Table S2).

### MTT assay

HEK-293T cells transfected with either pEFGP(N3) or pEGFP(N3)-SCGN constructs were seeded at a cell density of 15,000 cells/well in a 96-well plate. After 24 h, cells were incubated with increasing concentrations of rotenone (Sigma) (0, 100 nM, 500 nM, 1 μM, 10 μM, and 100 μM) for 24 h. Cells without any treatment and cells treated with 5% Triton X-100 were employed as controls. After 24 h of incubation, the cells were washed twice with PBS. Thereafter, 0.5 mg/ml of MTT was added to serum-free DMEM and incubated for 4 h at 37 °C in a CO_2_ incubator. Subsequently, MTT was removed, 100 μl of DMSO was added to dissolve MTT crystals and incubated for 30 min at 37 °C. Post incubation, the absorbance was recorded at 540 nm in an EnSpire Multimode plate reader and for% cell viability was calculated in the percentage of untreated control cells. Each group consisted of eight biological replicates.

### Cell culture–based luciferase assay

HEK-293T cells (0.2 × 10^6^) seeded in 6-well plates were transfected using lipofectamine LTX reagent (Invitrogen) by following the manufacturer’s protocol. Transfection was performed in incomplete media with firefly luciferase pGL3 vector (Promega luciferase assay system; Catalog No. E4030). Either only luciferase or luciferase with pEGFP(N3)-SCGN construct were cotransfected into cells. Luciferase and pEGFP(N3) vector cotransfection were used as controls. The transfection medium was replaced by DMEM complete media after 6 h. After 36 h of transfection, cells were incubated with 20 μg/ml cycloheximide for 30 min to suppress the expression of other chaperones. Subsequently, cells were subjected to heat shock at 42 °C for 45 min. Untreated cells were used as control. The luciferase activity for one set of cells was measured immediately after heat shock treatment, while for another set of cells after 4 h of recovery using EnSpire Multimode Plate Reader for reading time of 5 s with an initial delay time of 2 s in luminescence mode. All experiments were performed in triplicates. The percentage luciferase activity is calculated considering without heat shock (WHS) as a control for each group using the formula.

% Luciferase Activity=(Test/Control)∗100

### Small-angle X-ray scattering

SAXS data were collected as mentioned earlier on a Rigaku BioSAXS 2000 system at a wavelength of 1.54178 Å with 2D Kratky collimation, equipped with Rigaku HyPix-3000 hybrid pixel array detector attached to MicroMax 007 HF generator, operated at 40 kV and 30 mA (58). SAXS experiments were performed in either reducing (5 mM DTT) or oxidizing conditions (100 μM H_2_O_2_) with multiple concentrations of apo-SCGN ranging from 5 mg/ml to 15 mg/ml and in the presence of either 8 mM Ca^2+^ or 8 mM Mg^2+^. The protein solutions were prepared in 50 mM Tris, pH 7.5, 100 mM KCl buffer. The matched buffer for each sample was collected from flow through and used as a buffer blank.

Second set of experiments included Ca^2+^ titration (from 50 μM to 1 mM) with SCGN concentration at 7 mg/ml (with 5 mM DTT). It was an appropriate concentration at which the quality of data was good (signal/noise ratio). The sample and its matched buffer were exposed to X-ray for 60 min. The data were processed with the Automatic Data Analysis Pipeline (AAP), which is based on the ATSAS suite (version 2.7) and ATSAS online (Version 3.7) (59). The Guinier analysis was performed using RAW software (60).

All SAXS models were constructed using DAMMIN and GASBOR online tools (61, 62). Multi-FOXS analysis was carried out with apo crystal structure (PDB ID: 2be4) as well as with Ca^2+^-bound structure (PDB ID: 6jlh) of *D. rerio* SCGN.

### Isothermal titration calorimetry

The energetics of SCGN binding to substrates (α-synuclein and insulin) were studied at 30 °C on an ITC-200 instrument. SCGN samples (60 μM in the cell) and 600 μM α-synuclein (in the syringe) were prepared in Chelex-purified 50 mM Tris buffer, pH 7.5, and 100 mM KCl in the presence of 2 mM Ca^2+^. Insulin binding to SCGN in the absence/presence of Ca^2+^ were performed on a Microcal VP-ITC instrument at 30 °C in Chelex-purified 50 mM Tris, pH 7.5, 100 mM KCl. All experiments were performed using 1 mM insulin in the syringe and 30 μM SCGN. For Ca^2+^ free titrations, 100 μM EGTA was added both to SCGN and insulin, while for Ca^2+^ saturated titrations, 3 mM Ca^2+^ was added both to SCGN and insulin. The graphs obtained were buffer subtracted by titrating into α-synuclein or insulin with buffer under similar conditions. Data fittings were performed with the help of Origin 8 software.

### Dynamic light scattering

Dynamic light scattering measurements of SCGN (13 mg/ml) samples prepared in buffer (50 mM Tris, pH 7.5, and 100 mM KCl) were performed on an SZ-100 Horiba instrument SCGN (13 mg/ml) at room temperature with 20 accumulations each time. A buffer blank reading was used for buffer subtraction. Prior to analyses, the protein samples were centrifuged at 15,000 rpm for 15 min.

### MALDI analysis

SCGN samples (7 mg/ml) to be analyzed were mixed with equal volumes of a sinapinic acid matrix (1:1). The 2 μl mixed sample was loaded onto a MALDI plate and allowed to air dry. The MALDI plate was placed in a mass spectrometer to acquire the spectra. The m/z values were analyzed using the software MASCOT.

### Statistical analysis

All data were analyzed using GraphPad Prism 8 and expressed as mean ± standard deviation. A *p*-value of <0.05 was considered statistically significant between different test conditions determined by a two-sided Student’s *t* test for western and qRT-PCR experiments. Data from luciferase and MTT assay were analyzed, and *p*-value (<0.05 significance) was calculated by two-way ANOVA with Bonferroni multiple comparison test.

## Data availability

All data generated and analyzed during this study are included in this article.

## Supporting information

This article contains supporting information. Supporting Figures S1-S9 and Supporting Tables S1-S2.

## Conflict of interest

The authors declare that they have no conflict of interest with the content of this article.
