# Supplementary material for: Secretagogin is a Ca2+-dependent stress-responsive chaperone that may also play a role in aggregation-based proteinopathies
Source: J Biol Chem. 2022 Jul 21;298(9):102285. doi: 10.1016/j.jbc.2022.102285 (PMC9425029; doi:10.1016/j.jbc.2022.102285)
Supplement: Supporting Information [file mmc1.pdf]

Supporting Information

Secretagogin is a Ca^{2+}-dependent stress-responsive chaperone that may also play a role in aggregation-based proteinopathies

Amrutha H Chidananda[1], Radhika Khandelwal[1,2], Aditya Jamkhindikar[1], Asmita D Pawar,[1,3] Anand K Sharma[1,*], and Yogendra Sharma[1,2,3]*

[1]CSIR-Centre for Cellular and Molecular Biology (CCMB), Uppal Road, Hyderabad-500 007, India;
[2]Academy of Scientific and Innovative Research (AcSIR), Ghaziabad-201002, India;
[3]Indian Institute of Scientific and Education Research (IISER), Berhampur-760010, India.

***Correspondence**: Anand K Sharma, email: anand.sharma@hest.ethz.ch
or Yogendra Sharma, yogendra@iiserbpr.ac.in

Supporting Figures

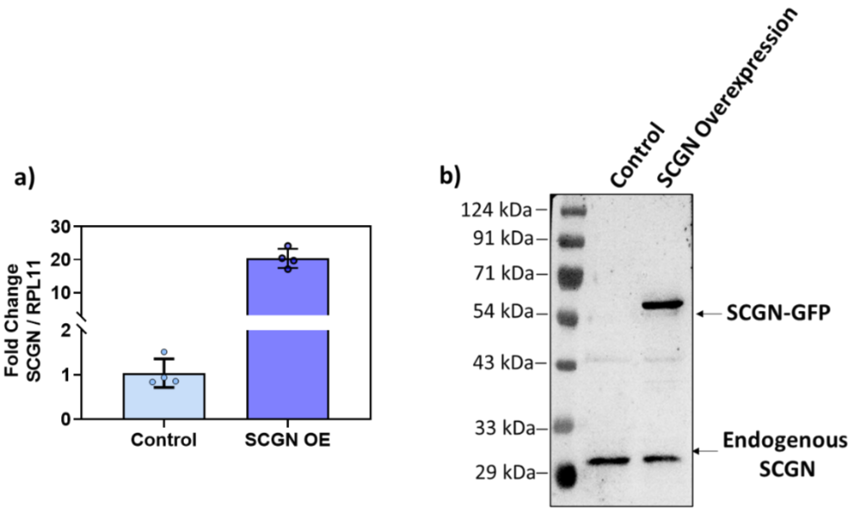

Figure S1: Validation of GFP-SCGN overexpression in transfected HEK-293T cell line. **(a)** mRNA expression in control and SCGN overexpressing (OE) HEK-293T cells quantified by qRT-PCR. **(b)** Western blot demonstrating overexpression of GFP-tagged SCGN (59 kDa) in HEK-293T cell line.

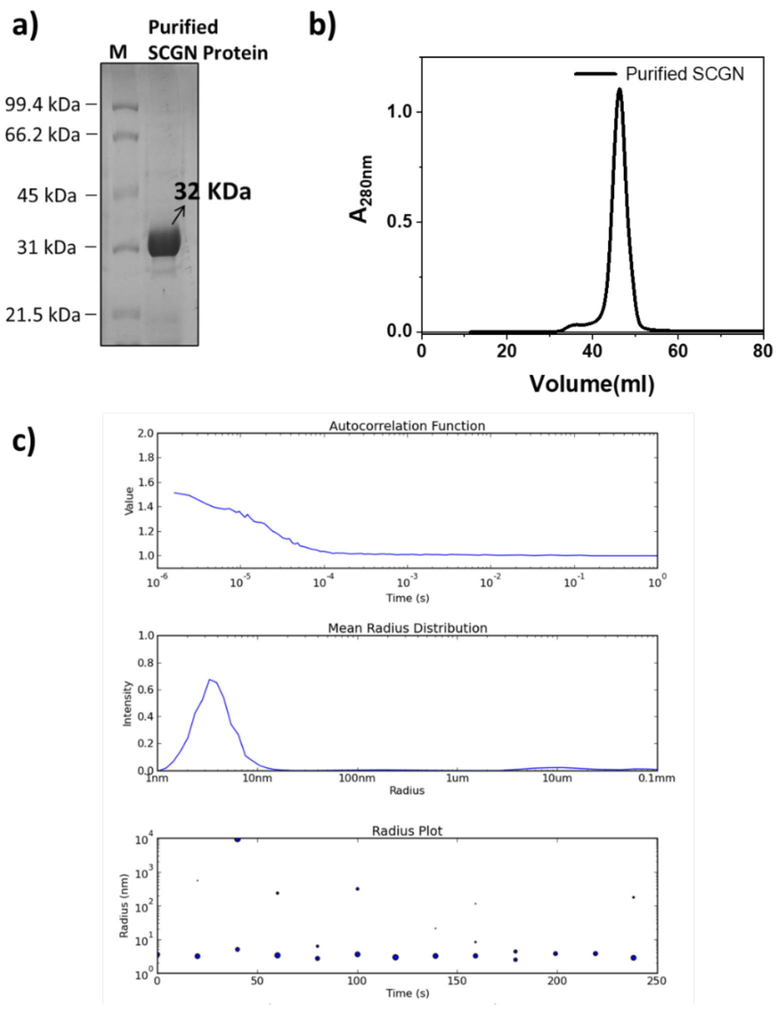

Figure S2: Protein quality assessment for SAXS experiments: (a) SDS-PAGE of SCGN purified from soluble fraction using Ni-NTA affinity chromatography and preparative size-exclusion chromatography. **(b)** Size-exclusion chromatogram of purified SCGN obtained on a preparative gel filtration column of Superdex 75pg. **(c)** Dynamic light scattering (DLS)-derived auto correlation function and mean size distribution parameters for assessment of protein homodispersity of purified and decalcified SCGN (13 mg/ml).

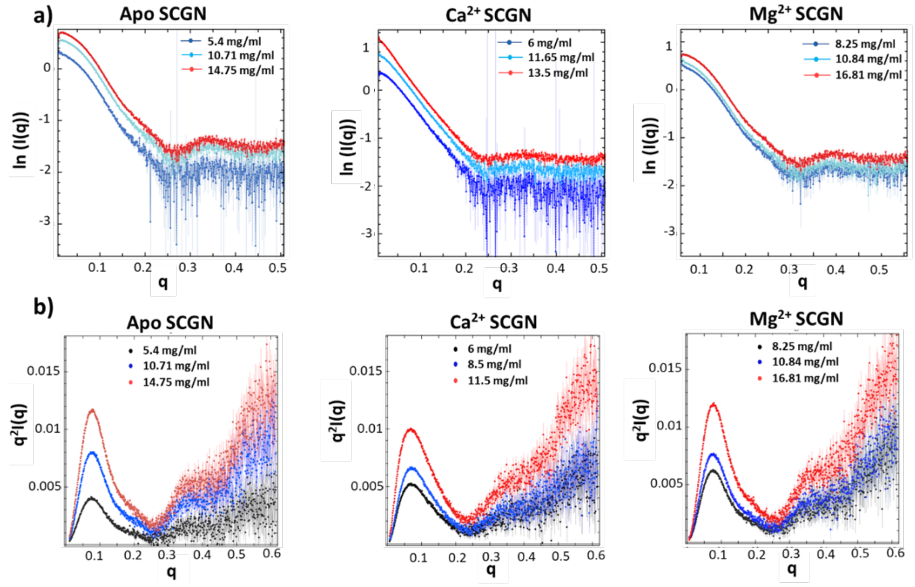

Figure S3: SAXS analysis of SCGN in presence of 8 mM Ca^{2+} or 8 mM Mg^{2+}. (a) Plot of log intensity of the X-ray scattering against the scattering vector for SCGN (q). **(b)** Data represented as a Kratky plot. Variation of q^2I with respect to q indicates that the protein is globular and folded. Protein concentrations used in each case are specified in the graph.

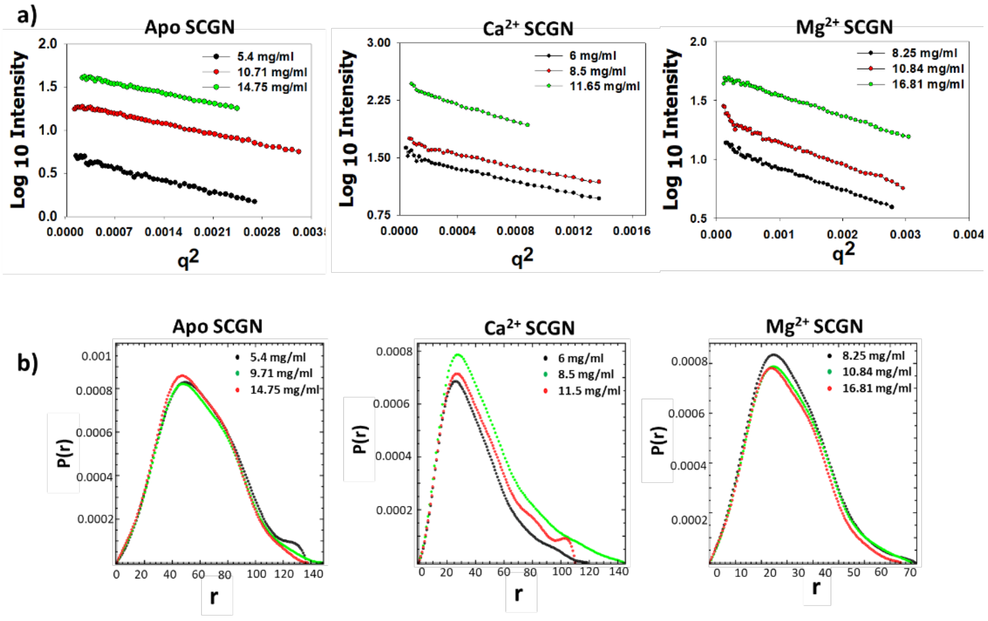

Figure S4: SAXS measurements and analysis. (a) The Guinier plots prepared using analyses with RAW software. **(b)** The pair distribution function [p(r)] for different concentrations of SCGN in the presence of either Ca^{2+} or Mg^{2+}.

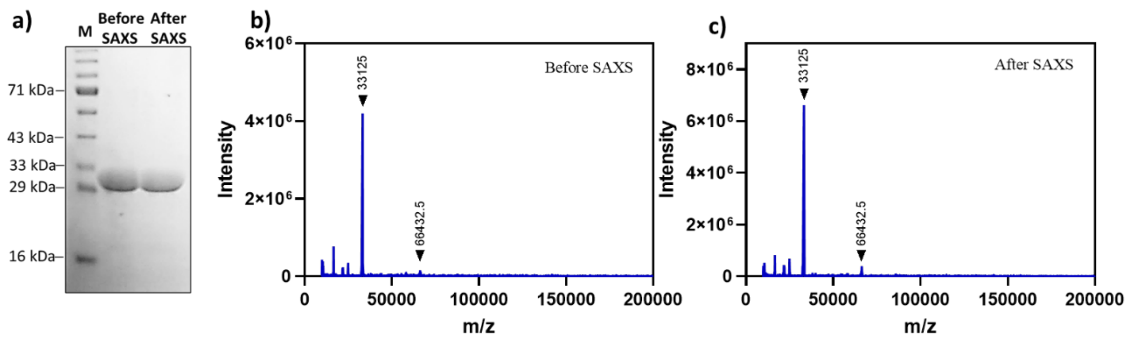

Figure S5: Assessment of protein quality and homodispersity of SCGN pre- and post-SAXS experiments. **(a)** SDS-PAGE analyses of SCGN before and after X-ray exposures. MALDI analyses of SCGN: **(b)** before, and **(c)** after SAXS experiments.

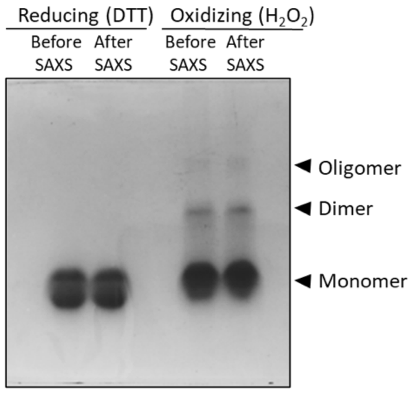

Figure S6: Redox-dependent oligomerization of SCGN. Native-PAGE of purified SCGN before and after SAXS experiment in presence of 2 mM Ca^{2+} in reducing (with 5 mM DTT) and in oxidizing (with 100 μM H_20_2) conditions. The protein quality remains unperturbed by the X-ray irradiation exposure during the SAXS experiment.

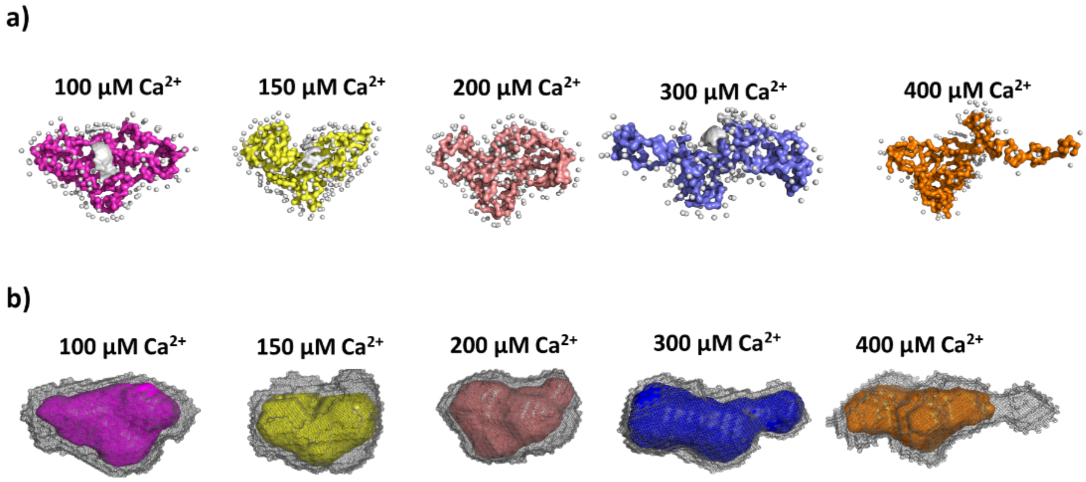

Figure S7: SAXS-derived structural models of SCGN in the presence of various Ca^{2+} concentrations (100, 150, 200, 300, and 400 μM). (a) The GASBOR *ab initio* model, and **(b)** DAMMIF models.

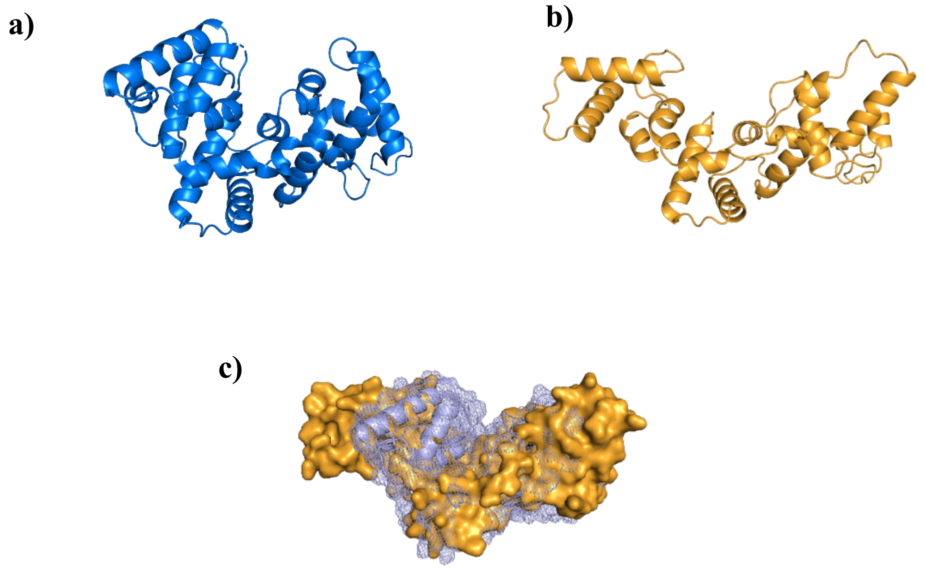

Figure S8: Comparison of Ca^{2+}-bound structures of SCGN and its comparison with calbindin D28K, a similar protein of hexa-EF-hand family. (a) Crystal structure of Ca^{2+}-bound calbindin D28K (PDB ID: 6fie). **(b)** Multi-FOXS model of Ca^{2+}-bound SCGN obtained by using SCGN crystal structure (PDB ID: 6jlh) and SAXS data. **(c)** Superimposing of Ca^{2+}-bound calbindin D28K (blue) and Ca^{2+}-bound SCGN (orange) using Pymol software. SCGN demonstrates greater conformational change in Ca^{2+}-bound form in comparison to Ca^{2+}-bound Calbindin D28k.

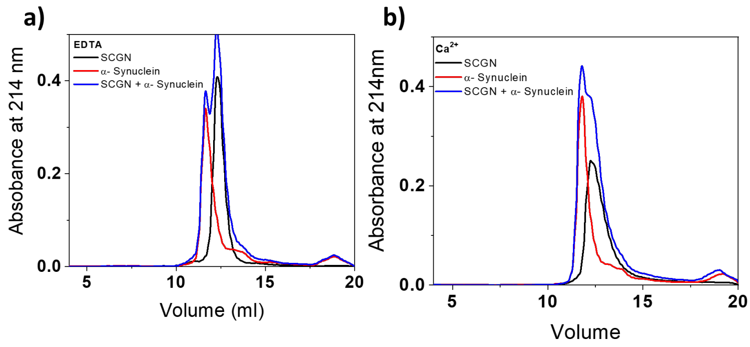

Figure S9: Analytical size-exclusion chromatography to assess the stability of SCGN and α-Synuclein complex in the presence of **(a)** 10 μM EDTA, and **(b)** 2 mM Ca^{2+}.

Table S1: SAXS-derived structural parameters for various concentrations of SCGN either without Ca^{2+} (apo) or with 8 mM Ca^{2+} or 8 mM Mg^{2+}. R_g and D_{max} values were computed from experimental data using ATSAS software.

SCGN (mg/ml)		I_0/c	$R_g^{Guinier}$ (Å)		$R_g^{P(r)}$ (Å)	D_{max} (Å)	Guinier Range		MW porod (kDa)
Apo	5.4	0.37	24	± 1.6	23.4	69	14	58	32.7
	10.71	0.35	22.6	± 0.2	22.9	74	11	62	32
	14.75	0.35	22.2	± 0.2	22.3	69	10	64	31.9
Ca^{2+}	8.50	0.54	35.2	± 0.6	37.7	144	8	37	49.8
	11.65	0.46	33.6	± 1.9	34.1	110	10	39	45.3
	13.50	0.86	44.6	± 4.0	47.5	223	6	20	66.3
Mg^{2+}	8.25	0.37	23.6	± 2.6	23.7	80	17	59	32.9
	10.84	0.37	23.9	± 3.9	23.9	80	17	59	32.9
	16.81	0.35	22.8	± 0.2	22.8	78	13	58	32.6

Table S2: List of rat-specific primers used for qRT-PCR.

NCBI Accession Number	Gene Name	Forward Primer (5'-3')	Reverse Primer (5'-3')
NM_201561.1	*Scgn*	AAGGTCATTTCCGAGGCTGAG	GAAGAGCCAGAATCCTTGCCA
NM_013083.2	*Grp78*	CCACCTATTCCTGCGTCGG	GCCACATACGACGGTGTGAT
NM_001004198.1	*Tbp*	GGCGGGGTCATGAAATCCA	AGTGATGTGGGGACAAAACGAG
NM_031971.2	*Hsp70*	GCAAGGCCAACAAGATCACCATCA	TCCTCTTTCTCAGCCAGCGTGTTA
NM_001025739.1	*Rpl11*	AGATACCGGCAACTTTGGCTT	CCTACCCAGCACCACATAGAAAT
NM_031144.3	*Actb*	TACTCTGTGTGGATTGGTGGC	TAAAACGCAGCTCAGTAACAGTC
